# Supplementary material for: The role of Central American barriers in shaping the evolutionary history of the northernmost glassfrog, Hyalinobatrachium fleischmanni (Anura: Centrolenidae)
Source: PeerJ. 2019 Jan 3;7:e6115. doi: 10.7717/peerj.6115 (PMC6321759; doi:10.7717/peerj.6115)
Supplement: Supplemental Information 1 — Spatial clustering and genetic diversity for mitochondrial DNA sequences per gene from Hyalinobatrachium fleischmanni. [file peerj-07-6115-s001.docx]

Ángela M. Mendoza, Wilmar Bolivar-G, Ella Vázquez-Domínguez, Roberto Ibañez, Gabriela Parra-Olea. *The role of Central American barriers shaping the phylogeographic structure of the northernmost glassfrog Hyalinobatrachium fleischmanni (Anura: Centrolenidae)*

**Supplementary Material 1.**

We performed separate analysis per gene to include all samples available avoiding the difficulties caused by missing data. First, we conducted a Bayesian analysis of population structure with BAPS v6.0 (Corander et al., 2008), using the spatial clustering of individuals, considering that the spatial prior may strengthen inferences for sparse molecular data. Considering that the pool of samples was slightly different for each gene and BAPS was very sensitive to missing data, BAPS analysis was run independently for each gene, with a maximum *k* value of 10 populations per analysis. Geneland and sPCA analyses as those generated for the concatenated sequences in the main text were also performed per gene. Additionally, a median-joining haplotype network for each gene was constructed using PopArt (French et al., 2014).

We finally defined genetically homogeneous regions as those obtained through all spatial, and non-spatial analyses for diversity index estimation. We calculated haplotype (h) and nucleotide diversity (π), globally and by region, with DNAsp (Libardo & Rozas, 2009). Additionally, the distribution of genetic variability at hierarchical levels was estimated using analysis of molecular variance (AMOVA). Genetic differentiation among all regions was again estimated based on the Nei's pairwise F_ST_ statistic with the *hierfstat* package (Goudet & Jombart 2015) and used corrected distances according to the K2P parameter (Kimura, 1980) in MEGA v.7 (Kumar, Strecher & Tamura 2016).

**Results**.

The BAPS results depicted six clusters for 16S, seven for COI and five for ND1. Although the number of clusters varied for the northern and southern regions, three clusters in western Chortis, Chorotega and eastern Chortis were consistently recovered. The 16S sequences separated the northern clusters, while the COI sequences could differentiate the southern clusters, east-west of the Andes range (Fig. S1.1).

Geneland analysis per gene showed six, seven and six clusters for 16S, COI and ND1, respectively. Both coding genes showed two clusters on both sides of the Andes range. Separation between the Choco and Chorotega samples was found in all cases (Fig. S1.1).

Overall, the haplotype networks for the three genes were concordant, with higher diversity and structure being revealed for COI and ND1 than for 16S. Four mitochondrial haplotype groups were detectable among the entire distribution (Fig. S3), where the concordance between the haplotype network and the species distribution suggested a deep pattern of geographic structuring and differentiation across the complete range. The SAP and Choco regions shared the same 16S haplotype but showed differences in the COI and ND1 coding genes.

The minimum genetic distance between regions was obtained for SAP-Choco and WPI (K2P = 0.007, 0.018 and 0.019 for 16S, COI and ND1 respectively) and the maximum between North American Pacific and WPI (K2P=0.029) for 16S, between Maya and SAP for COI (K2P= 0.078), and between SAP and Chorotega for ND1 (K2P=0.077) (Table S1.1). The *F_ST_* indices between regions ranged between 0.29 (SAP and Chorotega) and 0.9208 (WPI and Chorotega) in almost all combinations except between SAP and WPI for COI and ND1 (Table S1.1).

The AMOVA results for all genes indicated that 79-88% of the observed genetic variability was partitioned between regions, compared with 12-20% within regions (all P <0.001; Table S1.2).

When comparing the diversity per cluster, the Chorotega samples showed the highest haplotypic diversity for all genes, while the Maya populations exhibited the highest nucleotide diversity for coding genes. The Gulf population showed the lowest haplotypic diversity, and SAP exhibited the lowest nucleotide diversity (Table S1.3).

**References non-included in main text**

Corander J, Marttinen P, Sirén J, Tang J. 2008. Enhanced Bayesian modelling in BAPS software for learning genetic structures of populations. BMC bioinformatics 9(1):539

French N, Yu S, Biggs P, Holland B, Fearnhead P, Binney B, Fox A, Grove-White D, Leigh JW, Miller W, Muellner P, Carter P, 2014. Evolution of Campylobacter species in New Zealand, In: Sheppard SK, Méric G. eds. Campylobacter Ecology and Evolution. Caister Academic Press., Norfolk, pp. 221–240.

Goudet, J., & Jombart, T. (2015). hierfstat: estimation and tests of hierarchical F-statistics. R package version 0.04-22.

Kamvar ZN, Tabima JF, Grünwald NJ. 2014. Poppr: an R package for genetic analysis of populations with clonal, partially clonal, and/or sexual reproduction. PeerJ 2:e281 http://dx.doi.org/10.7717/peerj.281

Kimura M. 1980. A simple method for estimating evolutionary rates of base substitutions through comparative studies of nucleotide sequences. Journal of molecular evolution 16(2):111-120

Kumar S, Stecher G, Tamura K. 2016. MEGA7: molecular evolutionary genetics analysis version 7.0 for bigger datasets. Molecular biology and evolution 33(7):1870-1874

Libardo P, Rozas J, 2009. DnaSP v5: a software for comprehensive analysis of DNA polymorphism data. Bioinformatics 25(11):1451-1452

Table S1.1. Mean K2P distances within and among populations (lower) for all tree mitochondrial genes and F_ST_ indices (upper) per pair of populations detected.

| Block | Intra-population | SAP-Choco | WPI | Chorotega | Chortis | Maya | Gulf | Pacific |
| --- | --- | --- | --- | --- | --- | --- | --- | --- |
| 16S | | | | | | | |  |
| SAP-Choco | 0.002 | --- | 0.4507 | 0.8347 | 0.7467 | 0.8560 | 0.7347 | 0.9065 |
| WPI | 0.001 | 0.007 | --- | 0.8641 | 0.9591 | 0.6505 | 0.7226 | 0.8119 |
| Chorotega | 0.003 | 0.021 | 0.021 | --- | 0.8981 | 0.7693 | 0.7962 | 0.8822 |
| Chortis | 0 | 0.02 | 0.021 | 0.031 | --- | 0.4594 | 0.5762 | 0.6343 |
| Maya | 0.003 | 0.03 | 0.03 | 0.033 | 0.016 | --- | 0.3925 | 0.4923 |
| Gulf | 0.002 | 0.023 | 0.023 | 0.03 | 0.011 | 0.008 | --- | 0.4951 |
| Pacific | 0.003 | 0.028 | 0.029 | 0.041 | 0.019 | 0.022 | 0.015 | --- |
| COI | | | | | | | |  |
| SAP-Choco | 0.009 | --- | 0.0938 | 0.3840 | 0.5046 | 0.6661 | 0.4360 | 0.7214 |
| WPI | 0.008 | 0.018 | --- | 0.8761 | 0.5226 | 0.5219 | 0.8986 | 0.6609 |
| Chorotega | 0.006 | 0.054 | 0.044 | --- | 0.5266 | 0.6292 | 0.9120 | 0.7610 |
| Chortis | 0.005 | 0.071 | 0.067 | 0.06 | --- | 0.5158 | 0.4754 | 0.6003 |
| Maya | 0.015 | 0.078 | 0.068 | 0.071 | 0.052 | --- | 0.3509 | 0.6048 |
| Gulf | 0.006 | 0.068 | 0.062 | 0.067 | 0.047 | 0.038 | --- | 0.5880 |
| Pacific | 0.004 | 0.064 | 0.051 | 0.058 | 0.037 | 0.028 | 0.032 | --- |
| ND1 | | | | | | | |  |
| SAP-Choco | 0.01 | --- | 0.1793 | 0.2944 | 0.3648 | 0.5668 | 0.3608 | 0.6451 |
| WPI | 0.013 | 0.019 | --- | 0.9208 | 0.6556 | 0.6666 | 0.8510 | 0.8557 |
| Chorotega | 0.003 | 0.077 | 0.071 | --- | 0.6556 | 0.6666 | 0.8510 | 0.8557 |
| Chortis | 0.003 | 0.05 | 0.052 | 0.052 | --- | 0.5215 | 0.5548 | 0.6610 |
| Maya | 0.016 | 0.068 | 0.065 | 0.07 | 0.061 | --- | 0.3349 | 0.3723 |
| Gulf | 0.005 | 0.073 | 0.069 | 0.067 | 0.059 | 0.021 | --- | 0.5774 |
| Pacific | 0.017 | 0.074 | 0.071 | 0.065 | 0.059 | 0.032 | 0.03 | --- |

Table S1.2. AMOVA result for each mitochondrial gene per populations detected.

| **Source of variation** | **d.f.** | **Sum of squares** | **Mean Squares** | **Sigma** | **Percentage of variation** | **Φ statistics** |
| --- | --- | --- | --- | --- | --- | --- |
| **16S** | | | | | | |
| Between regions (ΦST) | 6 | 322.53 | 53.75 | 3.64 | 88.12 | 0.88 |
| Within regions (ΦCT) | 102 | 50.03 | 0.49 | 0.49 | 11.88 |  |
| **Total** | 108 | 372.57 | 3.45 | 4.13 | 100 |  |
| **COI** | | | | | | |
| Between regions (ΦST) | 6 | 1102.41 | 183.73 | 11.54 | 85.85 | 0.85 |
| Within regions (ΦCT) | 109 | 207.43 | 1.90 | 1.90 | 14.15 |  |
| **Total** | 115 | 1309.84 | 11.39 | 13.45 | 100 |  |
| **ND1** | | | | | | |
| Between regions (ΦST) | 6 | 676.78 | 112.80 | 15.42 | 79.65 | 0.79 |
| Within regions (ΦCT) | 47 | 185.17 | 3.94 | 3.93 | 20.34 |  |
| Total | 53 | 861.96 | 16.26 | 19.36 | 100 |  |

**Table S1.3.** Genetic polymorphism data for mitochondrial DNA sequences from the *Hyalinobatrachium fleischmanni* populations detected in this study.

|  | **N** | **h** | **S** | **Hd** | **π** | **Tajima’s D** | **Fu Fs** | **Rozas R Fs** |
| --- | --- | --- | --- | --- | --- | --- | --- | --- |
| **16S** | | | | | | | | |
| Total | 109 | 24 | 35 | 0.844 | 0.01554 | 0.16824 | -2.097 | 0.0906 |
| SAP-Choco | 47 | 9 | 9 | 0.414 | 0.00120 | -2.13240* | -8.151 | 0.0471 |
| WPI | 3 | 2 | 1 | 0.667 | 0.00159 | NA | NA | 0.4714 |
| Chorotega | 8 | 4 | 3 | 0.821 | 0.00266 | -0.1774 | -1.029 | 0.1804 |
| Chortis | 3 | 1 | 0 | 0 | 0 | NA | NA | NA |
| Maya | 20 | 4 | 7 | 0.431 | 0.0023 | -1.78918 | 0.122 | 0.1659 |
| Pacific | 5 | 2 | 3 | 0.4 | 0.00288 | -1.04849 | 1.688 | 0.4 |
| Gulf | 23 | 4 | 5 | 0.438 | 0.00156 | -1.52991 | -0.853 | 0.1307 |
| **COI** | | | | | | | | |
| Total | 116 | 63 | 110 | 0.979 | 0.04416 | 0.25275 | -11.326 | 0.1043 |
| SAP-Choco | 46 | 25 | 37 | 0.939 | 0.01270 | -0.70179 | -8.259 | 0.0812 |
| WPI | 3 | 3 | 4 | 1 | 0.00505 | NA | NA | 0.3118 |
| Chorotega | 7 | 6 | 8 | 0.952 | 0.00569 | -0.9744 | -2.238 | 0.2203 |
| Chortis | 8 | 4 | 6 | 0.785 | 0.00454 | 0.15875 | 0.522 | 0.1794 |
| Maya | 21 | 14 | 30 | 0.957 | 0.01388 | -0.47131 | -2.568 | 0.1098 |
| Pacific | 5 | 4 | 5 | 0.900 | 0.00417 | -0.56199 | -0.848 | 0.2408 |
| Gulf | 36 | 7 | 24 | 0.809 | 0.00601 | -1.80729* | 0.759 | 0.148 |
| **ND1** | | | | | | | | |
| Total | 54 | 45 | 141 | 0.99 | 0.04257 | -0.36248 | -9.584 | 0.1092 |
| SAP-Choco | 28 | 22 | 57 | 0.97 | 0.01341 | -1.31381 | -7.189 | 0.0764 |
| WPI | 3 | 3 | 5 | 1 | 0.00436 | NA | NA | 0.3399 |
| Chorotega | 3 | 3 | 3 | 1 | 0.00262 | NA | NA | 0.2722 |
| Chortis | 2 | 2 | 2 | 1 | 0.00262 | NA | NA | 0.5 |
| Maya | 8 | 7 | 42 | 0.964 | 0.01578 | -1.45862 | -0.108 | 0.1981 |
| Pacific | 2 | 2 | 13 | 1 | 0.01702 | NA | NA | 0.5 |
| Gulf | 8 | 6 | 14 | 0.892 | 0.00528 | -1.28604 | -0.94 | 0.1455 |

N = number of sequences, h = number of haplotypes, S = segregating sites, Hd = haplotype diversity, π = nucleotide diversity.

**
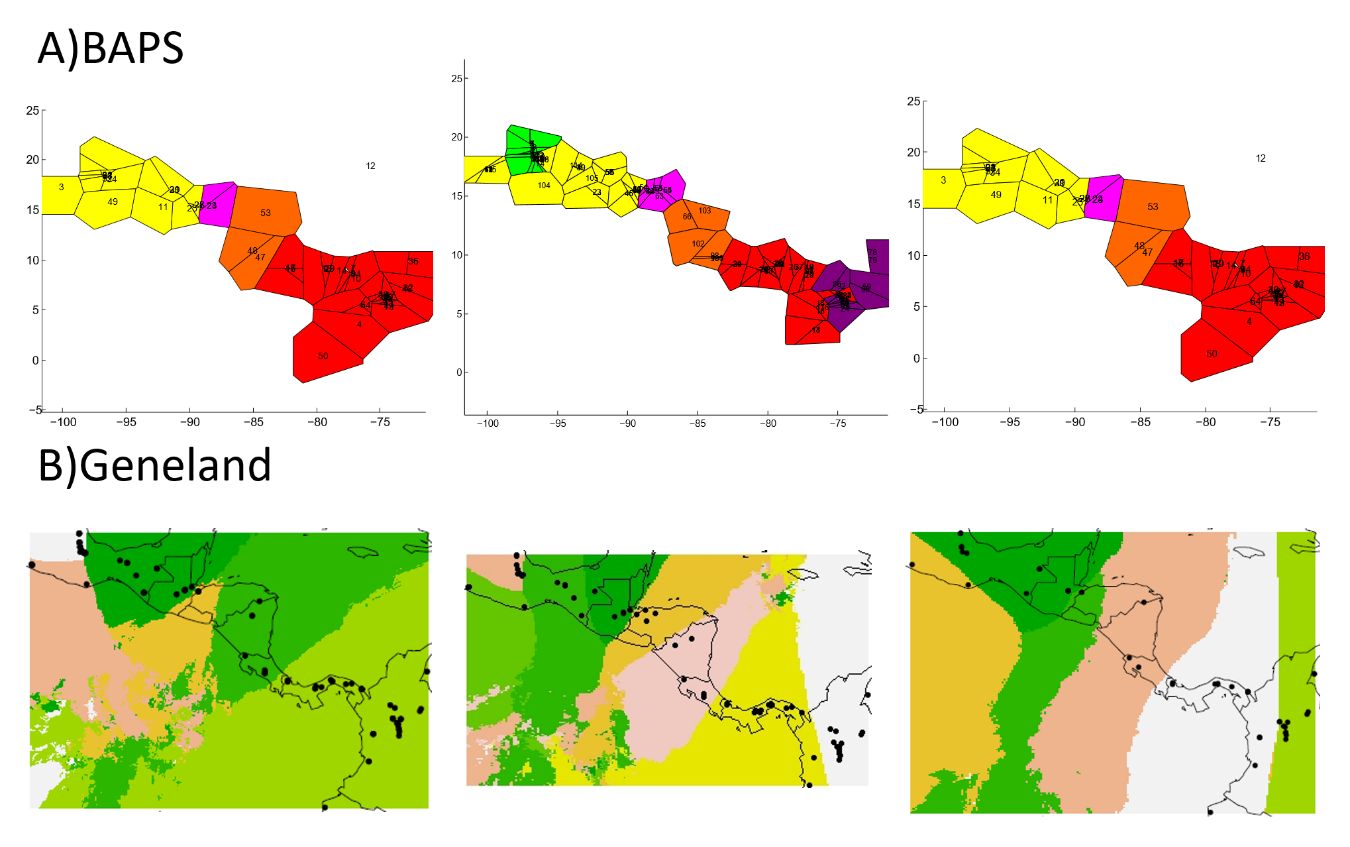
**

**Fig. S1.1.** Results of the Bayesian for *H. fleischmanni* population clustering A) BAPS and B) Geneland based on 16S (first column), COI (second column) and ND1 (third column) sequences.
