# Supplementary figures and images for: The role of Central American barriers in shaping the evolutionary history of the northernmost glassfrog, Hyalinobatrachium fleischmanni (Anura: Centrolenidae)
Source: PeerJ. 2019 Jan 3;7:e6115. doi: 10.7717/peerj.6115 (PMC6321759; doi:10.7717/peerj.6115)

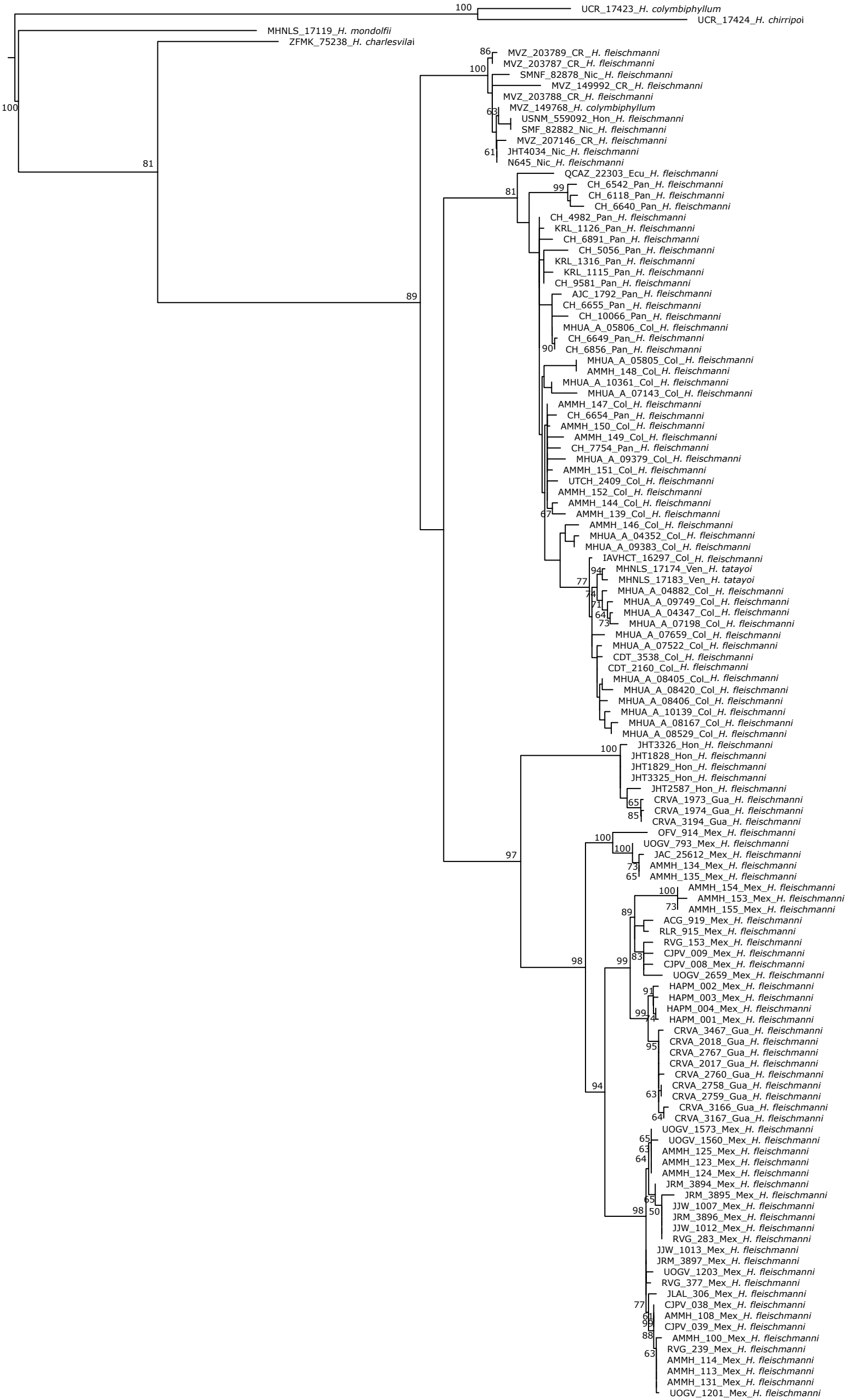

Supplement: Figure S1 — Phylogenetic relationships inferred from maximum-likelihood analyses (RAxML) of 3 genes sequenced from Hyalinobatrachium fleischmanni and sister species, with maximum-likelihood bootstrap support values >60 shown to the right of each node. For details see ‘Materials an Methods’ Section. [file peerj-07-6115-s003.pdf]

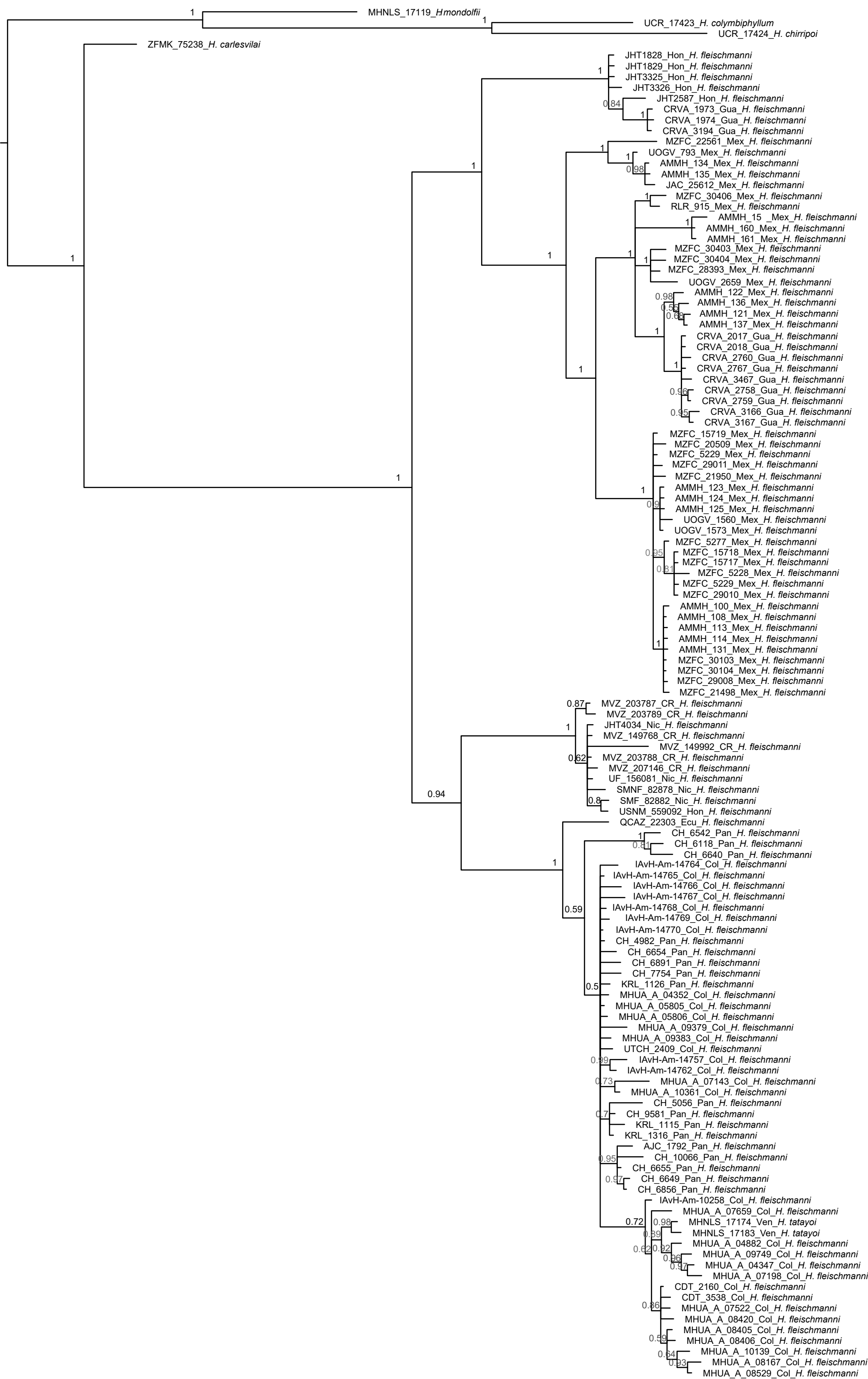

0.009

Supplement: Figure S2 — Phylogenetic relationships inferred from Bayesian analysis (Mr. Bayes) of 3 genes sequenced from Hyalinobatrachium fleischmanni and sister species, with maximum-likelihood bootstrap support values >0.50 shown to the right of each node. For details see Materials an Methods Section. [file peerj-07-6115-s004.pdf]

16S

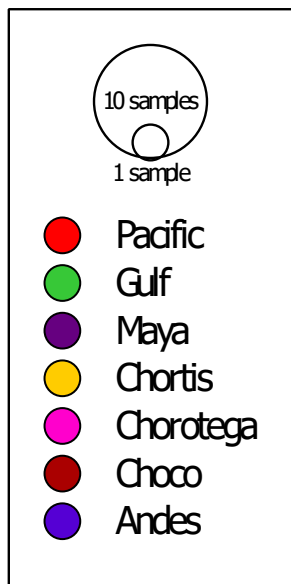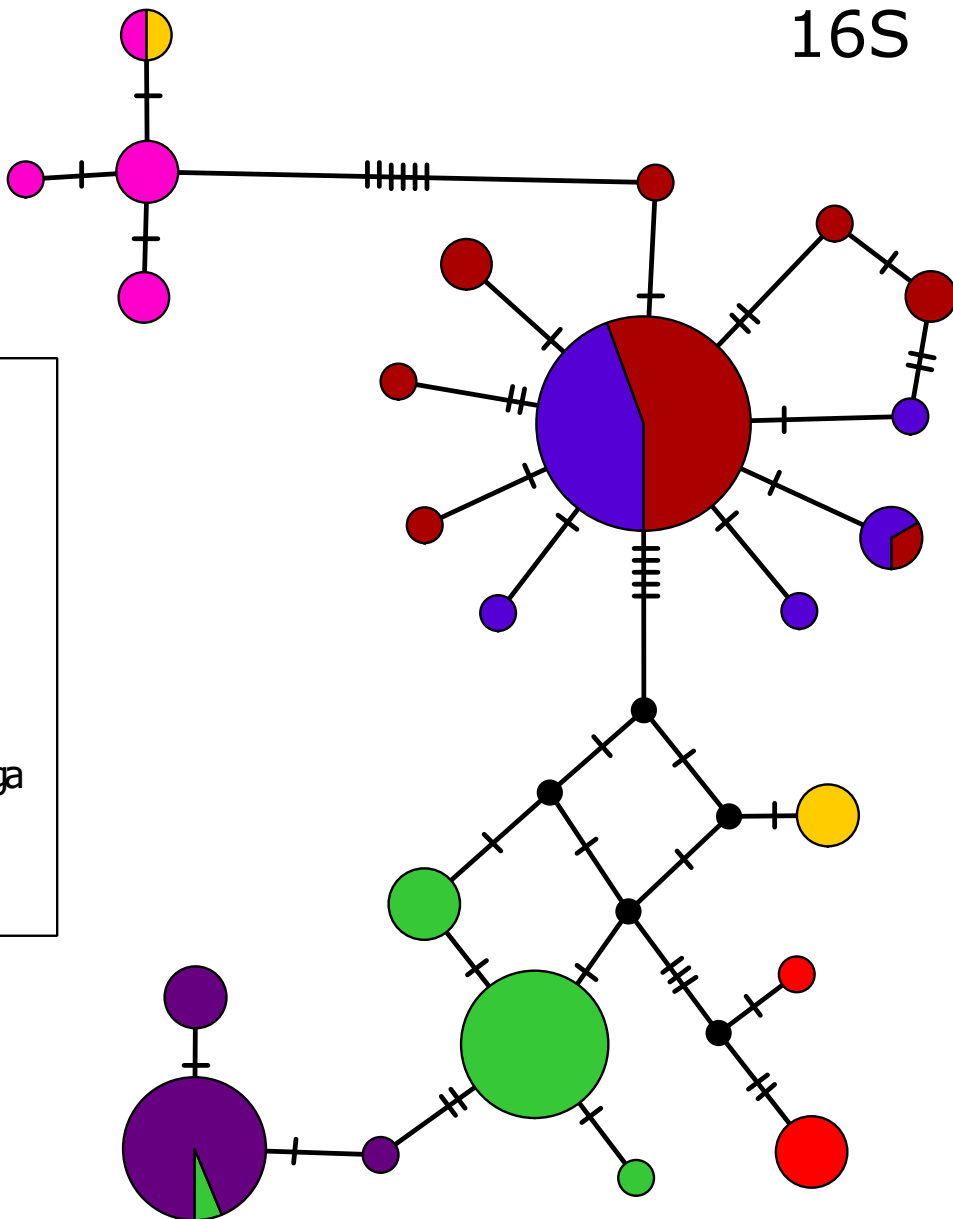

# COI

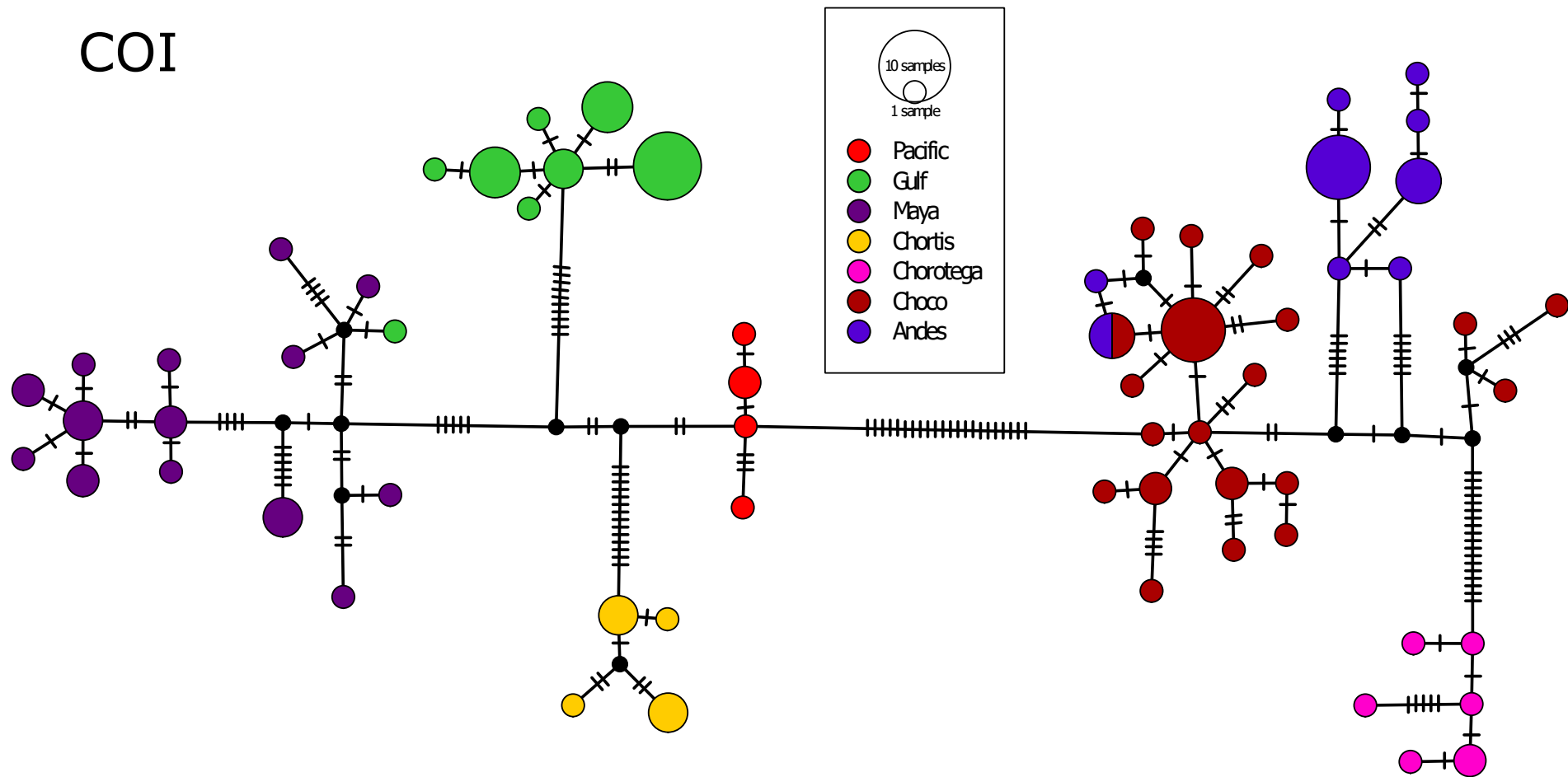

ND1

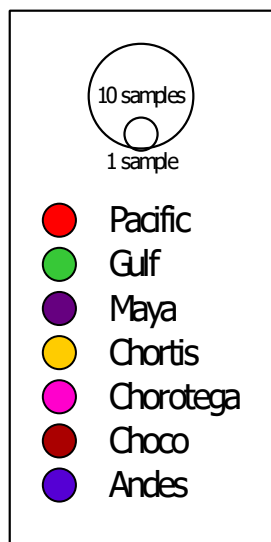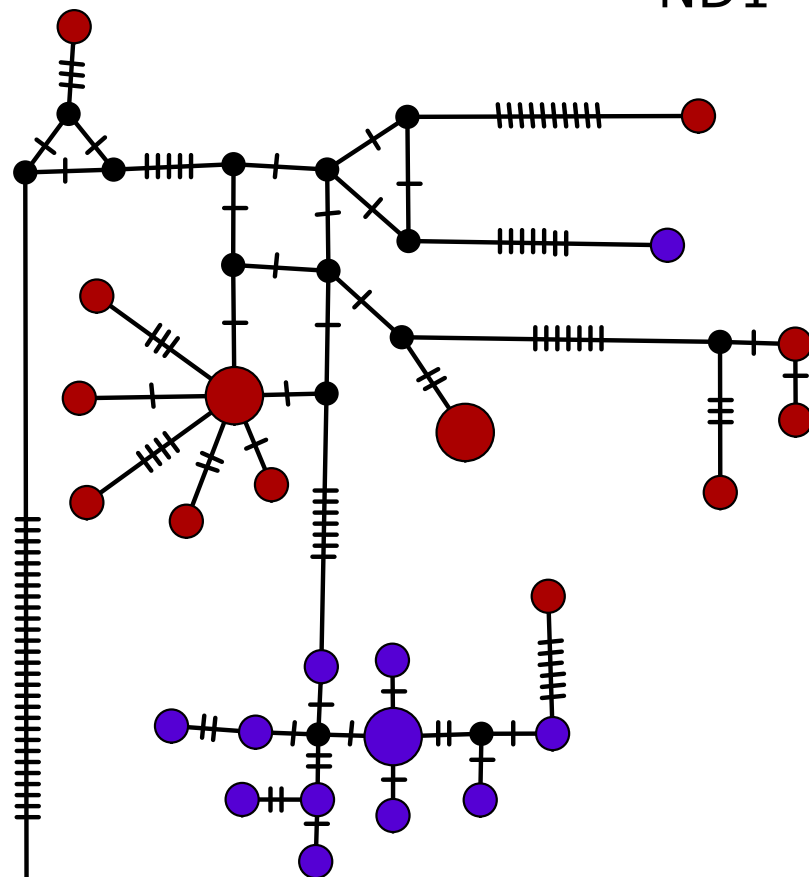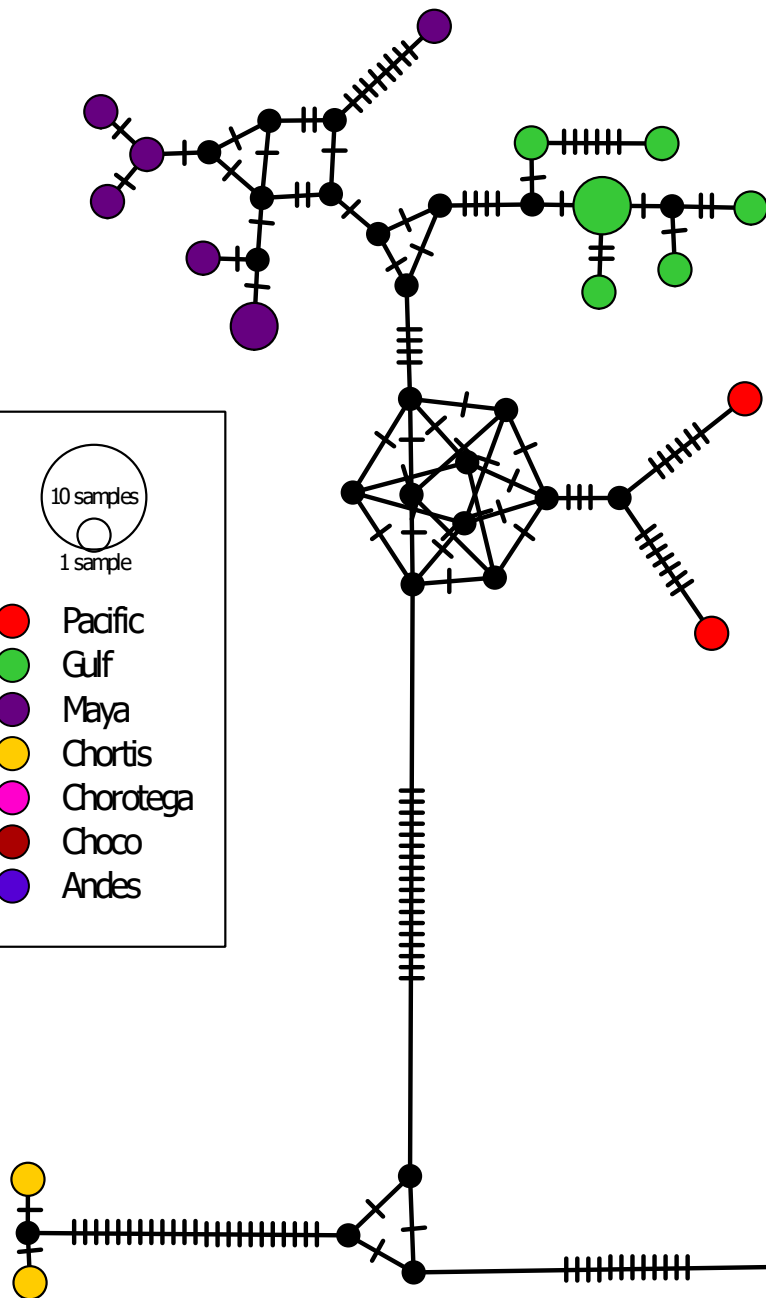

Supplement: Figure S3 — Haplotype networks of mitochondrial DNA haplotypes for 16S, COI and ND1 for H. fleischmanni. Hatch marks represent inferred mutational steps. The size of the circle is proportional to the number of individuals found for each haplotype. [file peerj-07-6115-s005.pdf]
